# CRISPR-Cas13a system: a novel approach to precision oncology

**DOI:** 10.20892/j.issn.2095-3941.2019.0325

**Published:** 2020-02-15

**Authors:** Junxia Zhang, Yongping You

**Affiliations:** ^1^Department of Neurosurgery, The First Affiliated Hospital of Nanjing Medical University, Nanjing 210029, China; ^2^Institute for Brain Tumors, Jiangsu Key Lab of Cancer Biomarkers, Prevention and Treatment, Jiangsu Collaborative Innovation Center for Cancer Personalized Medicine, Nanjing Medical University, Nanjing 210029, China

A paper titled “The CRISPR-Cas13a gene-editing system induces collateral cleavage of RNA in glioma cells”, recently published in *Advanced Science* by the Kang group, reports the promising application of the CRISPR-Cas13a system in cancer cells^1^. The CRISPR-Cas13a system not only knocked down the mRNA expression of the target gene EGFRvIII specifically but also induced a collateral RNA cleavage effect in U87-EGFRvIII glioma cells. Furthermore, the CRISPR-Cas13a system was found to effectively inhibit tumor growth and angiogenesis in an intracranial glioma tumor model.

The discovery of the CRISPR-Cas system, a bacterial adaptive immune system, has provided potent gene editing capability^2,3^. CRISPR-Cas systems are broadly divided into 2 classes on the basis of the construction of the interference module and further subdivided into 6 types. Class 1 CRISPR-Cas systems (types I, III, and IV) rely on multi-Cas protein complexes for interference, whereas class 2 systems (types II, V, and VI) use single effector proteins. The class 2 type II CRISPR-Cas9 system uses a DNA-targeting mechanism programmed through Watson–Crick RNA–DNA pairing. By taking advantage of precise genome modifications by the endogenous DNA repair machinery and high targeting efficiency, the CRISPR-Cas9 system has rapidly become a mainstream tool used for gene editing in a large body of research, including preclinical studies^4–7^.

Several novel Cas enzymes, such as the class 2 type VI effector proteins Cas13a (previously referred to as C2c2) and Cas13d, have been found to be CRISPR RNA (crRNA)-guided RNA-targeting CRISPR effectors^8,9^. Cas13a represents a class of enzymes with 2 separate RNase activities: RNA recognition and cleavage^10^. Furthermore, Liu and colleagues^11^ have identified 2 distant catalytic sites responsible for these activities: the REC lobe, with a Helical-1 domain, and the NUC lobe, with 2 HEPN domains. Upon formation of guide-target RNA duplexes, Cas13a is activated by triggering the HEPN1 domain to move toward the HEPN2 domain and subsequently bind and cleave target RNA bearing a complementary sequence^12^. Compared with another RNA editing method, RNAi, the CRISPR-Cas13a system has a higher knockdown efficiency in bacteria, plants, and mammalian cells^13,14^. Zhao et al.^15^ have shown that the CRISPR-Cas13a system can achieve greater than 90% knockdown efficiency in targeting KRAS-G12D mRNA while having no detectable effects on wild-type KRAS mRNA, and can lead to apoptosis and tumor growth inhibition in pancreatic cancer.

Interestingly, activated Cas13a induces the collateral cleavage of nearby nontargeted RNAs in a nonspecific manner and has an indirect growth suppression effect in bacteria. However, neither this nonspecific RNA cleavage activity nor cell growth inhibition had been observed in mammalian cells after the knockdown of targeted transcripts^8,13^. These novel results followed another important publication from the research group of Professor Chunsheng Kang^1^. In U87-EGFRvIII glioma cells, the authors confirmed the collateral cleavage effect of the CRISPR-Cas13a system through bulk and single-cell RNA sequencing analyses of global RNA expression^1^. In addition, the CRISPR-Cas13a system was found to inhibit glioma growth *in vitro* and *in vivo*^1^. Further experiments showed that the collateral effect occurred in LN229 glioma cells but not HEK293T cells. Several interesting questions relevant to the CRISPR-Cas13a system were raised in the current study. First, does the CRISPR-Cas13a system’s targeting of other genes result in the collateral RNA cleavage in glioma cells? Second, does the collateral cleavage effect occur in other cancer cells? Third, what is the intrinsic cleavage mechanism of CRISPR-Cas13a in glioma cells? Nevertheless, this is the first report of the collateral RNA effect in mammalian cells.

The activation of Cas13a and subsequent cleavage of trans-RNAs for every target RNA detected can enable potent signal amplification. Recently, Gootenberg et al.^16,17^ have established a Cas13a-based molecular detection platform called Specific High-Sensitivity Enzymatic Reporter UnLOCKing (SHERLOCK) to detect DNA and RNA *in vitro*, such as specific strains of Zika virus and low-frequency mutations in human cell-free tumor DNA. Bruch and colleagues^18^ have reported a CRISPR-Cas13a-powered electrochemical microfluidic biosensor system to detect miR-19b in serum samples from patients with brain cancer. Within 9 min, a detection limit of 10 pm can be achieved by measuring a volume less than 0.6 µL. The authors demonstrated the feasibility of the electrochemical CRISPR-powered system as a low-cost, easily scalable, and rapidly detectable tool for nucleic acid-based diagnostics.

In terms of mechanism and functionality, there are notable differences between CRISPR-Cas13a and CRISPR-Cas9 (**Table 1**). CRISPR-Cas13a cannot be concluded to be better or worse than CRISPR-Cas9, because both have different signatures and applications. We anticipate a new era of cancer treatment in which the CRISPR-Cas13a system will serve as an important bridge between bench and bedside. However, understanding of the CRISPR-Cas13a system remains in its infancy (**Figure 1**). The CRISPR-Cas13a system presents a promising platform for rapid nucleic acid detection with attomolar sensitivity and single-base mismatch specificity. For example, tumor-derived DNA and RNA in the blood, cerebrospinal fluid and other body fluids can be rapidly detected and applied to cancer diagnosis and monitoring and therapy guidance. Recent development and ongoing work demonstrate the remarkable potential of the CRISPR-Cas13a system in cancer therapies as an RNA editing tool^1,15,19^. Nevertheless, numerous problems still lie ahead in the translation to cancer therapy. The key challenge will be to adapt and design efficient delivery systems for target tissues. Currently, compared with other viral and nonviral vectors, recombinant AAVs are the leading platform for *in vivo* delivery of gene therapies^20^. Although the clinical success of recombinant AAV gene therapy is encouraging, the shortcomings of this gene delivery platform must be addressed. The immune response and cytotoxicity of the CRISPR-Cas13a system should also be evaluated.

Despite these challenges, the CRISPR-Cas13a system holds promise for the development of basic and clinical research in precision oncology. In the near future, we expect to see substantially improved understanding of the intricacy and diversity of CRISPR-Cas13a biology and clinical advancements in CRISPR-Cas13a technologies in the caner field.

## Figures and Tables

**Figure 1:**
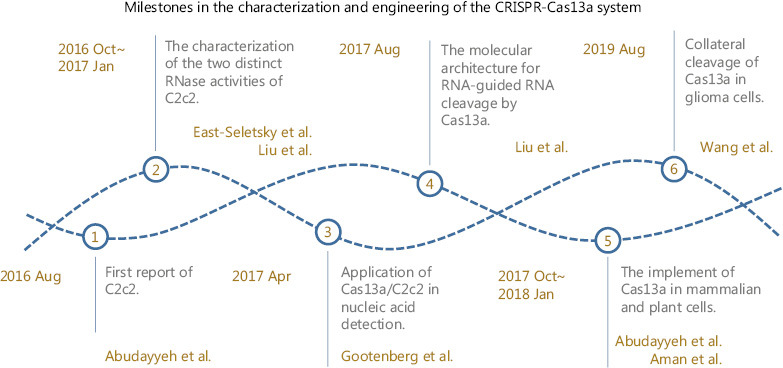
Key studies on the characterization and engineering of the CRISPR-Cas13a system.

**Table 1:** Comparison of CRISPR-Cas9 and CRISPR-Cas13a

Item	CRISPR-Cas9	CRISPR-Cas13a
Subtype	Type II	Type IV
Guide RNA	tracrRNA and crRNA	crRNA
Target substrate preference	PAM	PFS
Nuclease domain	HNH and RuvC	HEPN
crRNA processing	RNase III	Helical-1 domain
Applications	DNA targeting, tracking, editing RNA targeting, tracking	DNA detection RNA targeting, tracking
